# Emerging Roles and Potential Applications of Non-Coding RNAs in Cervical Cancer

**DOI:** 10.3390/genes13071254

**Published:** 2022-07-15

**Authors:** Deepak Parashar, Anupam Singh, Saurabh Gupta, Aishwarya Sharma, Manish K. Sharma, Kuldeep K. Roy, Subhash C. Chauhan, Vivek K. Kashyap

**Affiliations:** 1 Department of Obstetrics and Gynecology, Medical College of Wisconsin, Milwaukee, MI 53226, USA; 2 Department of Biotechnology, GLA University, Mathura 281406, Uttar Pradesh, India; anupamsinghsoul@gmail.com (A.S.); saurabh.gupta@gla.ac.in (S.G.); 3 Sri Siddhartha Medical College and Research Center, Tumkur 572107, Karnataka, India; aishwarya.sharma0909@gmail.com; 4 Department of Biotechnology, IP College, Bulandshahr 203001, Uttar Pradesh, India; mny2096@gmail.com; 5 Department of Pharmaceutical Sciences, School of Health Sciences and Technology, UPES, Dehradun 248007, Uttarakhand, India; kuldeep.roy@ddn.upes.ac.in; 6 Department of Immunology and Microbiology, School of Medicine, University of Texas Rio Grande Valley, McAllen, TX 78504, USA; subhash.chauhan@utrgv.edu; 7 South Texas Center of Excellence in Cancer Research, School of Medicine, University of Texas Rio Grande Valley, McAllen, TX 78504, USA

**Keywords:** cervical cancer, non-coding RNAs, diagnosis, prognosis, therapeutics, regulation of gene expression

## Abstract

Cervical cancer (CC) is a preventable disease using proven interventions, specifically prophylactic vaccination, pervasive disease screening, and treatment, but it is still the most frequently diagnosed cancer in women worldwide. Patients with advanced or metastatic CC have a very dismal prognosis and current therapeutic options are very limited. Therefore, understanding the mechanism of metastasis and discovering new therapeutic targets are crucial. New sequencing tools have given a full visualization of the human transcriptome’s composition. Non-coding RNAs (NcRNAs) perform various functions in transcriptional, translational, and post-translational processes through their interactions with proteins, RNA, and even DNA. It has been suggested that ncRNAs act as key regulators of a variety of biological processes, with their expression being tightly controlled under physiological settings. In recent years, and notably in the past decade, significant effort has been made to examine the role of ncRNAs in a variety of human diseases, including cancer. Therefore, shedding light on the functions of ncRNA will aid in our better understanding of CC. In this review, we summarize the emerging roles of ncRNAs in progression, metastasis, therapeutics, chemo-resistance, human papillomavirus (HPV) regulation, metabolic reprogramming, diagnosis, and as a prognostic biomarker of CC. We also discussed the role of ncRNA in the tumor microenvironment and tumor immunology, including cancer stem cells (CSCs) in CC. We also address contemporary technologies such as antisense oligonucleotides, CRISPR–Cas9, and exosomes, as well as their potential applications in targeting ncRNAs to manage CC.

## 1. Introduction

Cervical cancer (CC) is the fourth most frequently diagnosed cancer in women with substantial geographical variation in CC morbidity and mortality [1]. CC was accounted to cause approximately 604,000 new cases and 342,000 deaths worldwide in the year 2020 [1]. CC develops in the uterine cervix epithelium, notably at the squamo columnar junction, interface of the ectocervix and endocervix, which is a hotspot for metaplastic activity. Squamous cell carcinomas (SCC) and adenocarcinomas (ADC) are the most frequently diagnosed kinds of CC, accounting for approximately 80–90% and 10–15% of all cervical malignancies [2]. Adenosquamous carcinoma (ADSC) is a rare type of CC [2].

Human papillomavirus (HPV) infections, the most prevalent sexually transmitted infection, is responsible for causing cervical carcinogenesis [3,4]. The viral DNA gets integrated into the host DNA after a long-term high-risk HPV (HR-HPV) infection, and consequently, cervical epithelial cells become malignant, resulting in CC [5,6]. Moreover, precancerous mutations in the cervix lead to the establishment of CC. Additionally, the lag between infection and carcinogenesis is a major factor as to why CC has become a ravaging disease for women. Fortunately, earlier detection, awareness, and effective treatment of CC have been shown to considerably reduce both the morbidity and mortality rate in women. Effective monitoring and vaccination campaigns have resulted in a substantial drop in the CC fatality rate in developed countries over the last four decades [7]. The Papanicolaou (PAP) smear test, visual inspection with acetic acid (VIA), liquid- based cytology (LBC), and HPV testing for HR-HPV strains are some of the current screening approaches utilized for detecting cancer in the early stages [8]. Furthermore, venereal diseases, long-term oral contraception, reproductive factors, and behavioral issues such as smoking, drinking, and obesity have all been identified as CC risk factors [6,9].

Chemotherapy, radiation, and surgery are all available treatments for CC, but none of these improve patient survival rates and can result in serious negative effects. Despite all these advances in the detection and prevention of CC, it remains “a worldwide health crisis”, particularly in undeveloped and emerging countries [10,11]. In spite of recent breakthroughs, CC has a poor long-term prognosis due to its resilience and relapsing nature. This necessitates the development of new biomarkers for tracking CC progression, which also serve as putative targets for diagnostic and curative purposes. Expression profiling of several ncRNAs has been shown to be correlated with cancer progression, onset, metastases, and invasion and has emerged as a novel prognostic and diagnostic biomarker in cervical carcinoma [5]. This article provides a comprehensive overview of the function and potential application of ncRNAs in CC.

## 2. Classification and Biogenesis of ncRNAs

### 2.1. Classification

According to the literature, ncRNAs can be classified according to their structure, function, biogenesis, localization, and interaction with DNA or protein-coding mRNAs [12,13]. The discovery of the order of activities in the passage of genetic information stored in DNA to working biological processes via proteins has been dubbed the central dogma of molecular genetics by Francis Crick in 1958, and it was a watershed moment in molecular biology [14]. With the emergence of novel technologies and rigorous next-generation sequencing, large international consortiums such as the Functional Annotation of the Mammalian Genome (FANTOM) and the Encyclopedia of DNA Elements (ENCODE) have explained ubiquitous transcription as ~98% of DNA is transcribed into RNA, and only ~2% of that RNA is translated into protein [15,16,17]. Therefore, in the world of cellular communication, RNA is divided into two distinct types: coding RNAs and ncRNAs. The major chunk of transcribed DNA, i.e., ncRNA, was earlier thought to be evolutionary garbage since it lacked the ability to code for protein, and protein-coding RNA, which is a considerably smaller portion of RNA [18,19].

**Figure 1:**
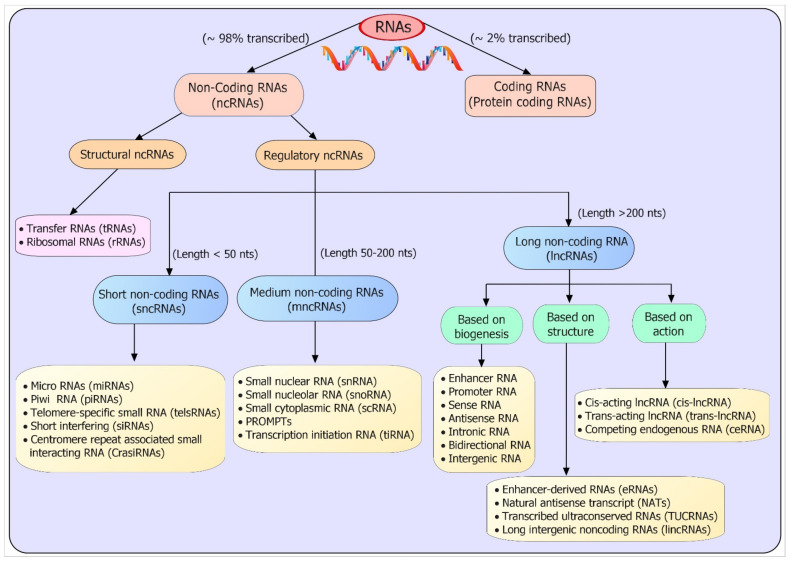
Non-coding RNA (ncRNA) classification. The schematic shows that ncRNAs are divided in to two major categories, such as structural and regulatory ncRNAs. Regulatory ncRNAs are further divided into small (<50 nts length), medium (50–200 nts length), and long non-coding RNAs (>200 nts length), according to their length. LncRNAs are further classified according to their structure, biogenesis, and mechanism of action. See the text for more details.

NcRNAs are basically divided into two domains: structural ncRNAs and regulatory ncRNAs. Structural ncRNAs include transfer RNAs (tRNAs) and ribosomal RNAs (rRNAs). Regulatory ncRNAs are further classified into small (length < 50 nts), medium (length 50–200 nts), and long non-coding RNAs (lncRNAs) (length > 200 nts), based on transcript length (Figure 1) [20,21,22]. Furthermore, microRNAs (miRNAs), small interfering RNAs (siRNAs), piwi-interacting RNAs (piRNAs), cisRNA, and telomere-specific small RNAs (tel-sRNAs) belong to the category of short non-coding RNA (sncRNAs), having a transcript size ranging between 20–50 nucleotides, and similarly, small nucleolar RNA (snoRNA), prompts, tiRNA, small nuclear RNA (snRNA), and small cytoplasmic RNA (scRNA) can be categorized as medium ncRNAs with a transcript length between 50–200 nucleotides [23,24]. LncRNAs regulate transcripts possessing a size greater than >200 nucleotides. Furthermore, lncRNA could be divided into three main categories. The first category is based on biogenesis of lncRNAs such as intronic, intergenic, sense, antisense, bidirectional, and promoter and enhancer lncRNAs, whereas the second category is based on the mechanism, such as cis-regulatory RNA (cis-RNA), trans-RNA, and competing endogenous RNAs (ceRNAs), long intergenic non-coding RNA (lincRNAs), while the third category is based on structure, such as natural antisense transcripts (NATs), enhancer-derived RNAs (eRNA), and circular RNA (circRNA) [25,26,27].

### 2.2. Biogenesis of ncRNAs

**Figure 2:**
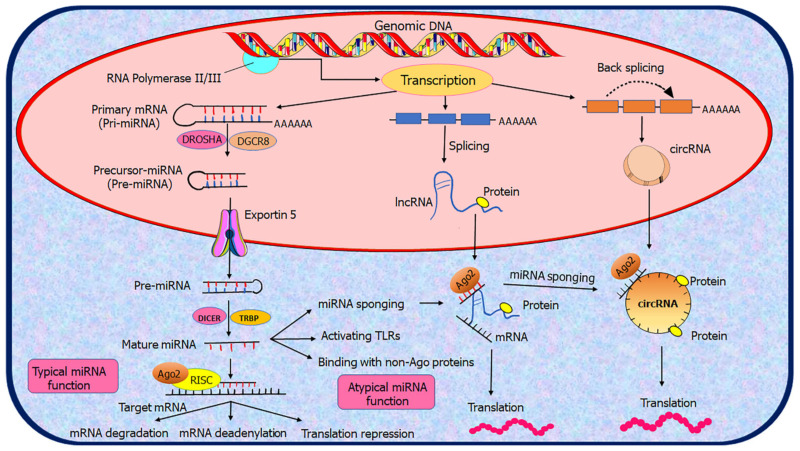
Overview biogenesis and function of ncRNAs in CC cells. MicroRNAs (miRNAs), long non-coding RNAs (lncRNAs), and circular RNAs (circRNAs) are presented along with their fundamental biogenesis and main functional mechanisms. The details are described in the text.

The biogenesis of ncRNAs is predicated on their characteristics, which are comparable to those of mRNAs. NcRNA play a crucial function in several prospects of human development and diseases [28]. Addressing ncRNA biogenesis is important not only for distinguishing it from the rest of the RNAs but also for assessing its functional relevance [29]. Across the human genome, several genes participate in the generation of various types of ncRNAs [30]. Transcription, nucleosomal maturation, exportation towards the cytoplasm for processing, and production of functional RNA are all quintessential parts of the biogenesis process. RNA polymerase II/III transcribes polycistrons, producing large progenitors (pri-miRNA: hairpin loop structure; 5′capping; 3′polyadenylation) (Figure 2) [31]. After that, it passes through two steps of processing: The microprocessor (DGCR8) identifies and controls the breaking of pri-miRNA via Drosha’s, resulting in the emergence of pre-miRNA, which is then translocated from the nucleus to the cytoplasmic region via RAN-GTP and Exportin-5 (XPO5) protein. Furthermore, in the cytoplasm, Dicer acting as RNase III endonuclease chops the progenitor molecule present more towards the terminal end, releasing an RNA duplex that interfaces with Argonaute proteins (AGO-2) present in collaboration with RISC (miRNA-induced silencing complex) [31,32]. However, lncRNA biogenesis proceeds under the influence of the type of cell and phase-specific stimulation governs it [33].

Multiple DNA components in eukaryotic genomes, including enhancers, promoters, and intergenic portions, transcribe distinct kinds of lncRNAs [34]. The principle processes carried out during biogenesis include cleavage by Ribonuclease P (RNase P) to create mature ends, production of snoRNA and small nucleolar ribonucleoprotein (snoRNP) complexes, capping at their ends, and the formation of circular structures [35]. During the synthesis of particular lncRNAs, distinctive sub-nuclear structures known as “paraspeckles” have recently been discovered [36]. Overall, the processes of biosynthetic pathways and regulation of unique ncRNAs are not entirely comprehended. However, we will gain a better understanding of their genesis and applications in the coming years by using a variety of techniques such as ChiRP-Seq (Chromatin Isolation by RNA Purification), RNA structure mapping, crosslinking immunoprecipitation (CLIP), targeted genome engineering with CRISPR–Cas9 and advanced genetic monitoring, ribosome profiling, and phylogenetic lineage tracing [37].

## 3. Functional Roles and Mechanisms of Action of ncRNAs

### 3.1. Biological Function of ncRNAs

**Figure 3:**
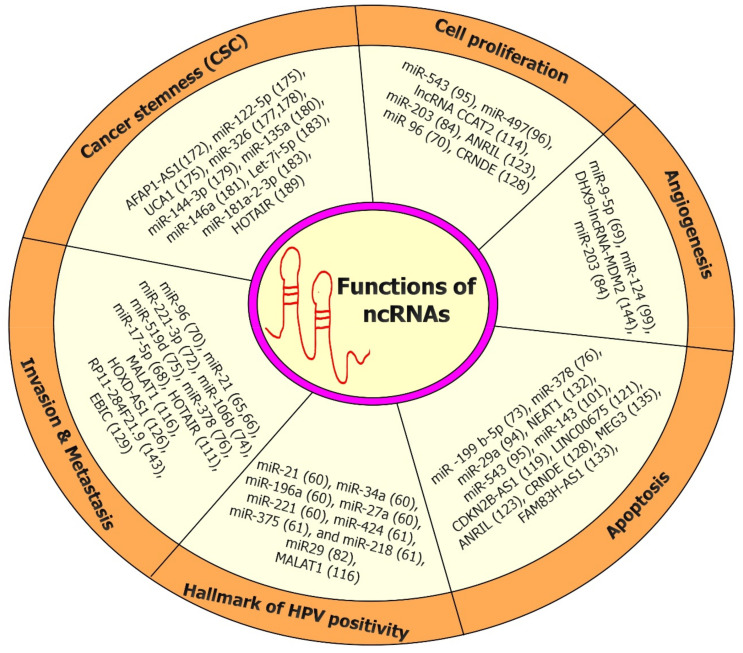
Different biological function of ncRNAs in CC. NcRNAs play a crucial role in the regulation of cell proliferation, cell cycle, cell migration and invasion, epithelial–mesenchymal transition (EMT), and angiogenesis in CC.

The biological functions of ncRNAs have been progressively explained, including the regulation of gene expression at the transcriptional and translational levels; instructing DNA synthesis or gene rearrangement; and guarding the genome from foreign nucleic acids [38]. Several recent studies have indicated that ncRNAs are crucial in carcinogenesis by controlling the expression of cancer-associated genes [31,39,40,41]. Mechanistically, lncRNAs govern gene expression primarily by functioning as transcription factors, controlling chromatin remodeling, or actively contributing to posttranscriptional regulation as ceRNAs [42,43,44]. MiRNAs, on the other hand, control gene expression at the posttranscriptional level via RNA interference and frequently attach to the 3′-untranslated region (3′UTR) of protein-coding mRNAs and to the (5′UTR) or coding sequence [45,46,47,48]. Furthermore, through complementary binding with aimed genes, a few tRNA fragments (TRFs) and tRNA-derived stress-induced RNAs (tiRNAs) may contribute to gene regulation and gene silencing, following a mechanism identical to that of miRNA [49]. CircRNAs primarily operate as ceRNAs and control gene expression at three distinct levels, including epigenetic, transcriptional, and posttranscriptional by sponging several miRNAs (Figure 3) [50].

### 3.2. Mechanisms of Action

LncRNAs and miRNAs are structurally similar, and both play crucial roles in the modulation of gene expression. By recruiting multi-subunit chromatin modifying complexes to the DNA molecule (chromatin modulation), some ncRNA regulate multiple biological phenomena such as transcription, nucleosome orientation, chromatin labelling, or histone modifications [19]. All of these modulate gene expression of target genes. Certain miRNAs show interaction with a specific region of the gene promoter. For instance, miR-24-1 acts as an enhancer trigger to stimulate enhancer RNA (eRNA) expression, alters histone modification, and increases the enrichment of p300 and RNA Pol II at the enhancer locus [51]. Some ncRNAs act via splicing regulation and influence disease progression and essential physiological functions by adhering to distinct protein networks that regulate gene expression. Spliceosomes are generated by snRNA and proteins, and they are responsible for the splicing mechanism [19]. For example, SHARP, SAF-A, and LBR are key proteins that are associated with Xist lncRNA for Xist-mediated transcriptional silencing on the X chromosome [52]. Some act via crosstalk in proteomics. Such a mechanism leads to the production of ceRNA and governs translation, transcription, epigenetics, pathological, and physiological processes, exemplified by certain lncRNAs playing a quintessential role during oncogenesis and tumor suppressor cascade and by miR-124, miR-375, and let-7b, which inhibit erbB2/erbB3 to cure breast cancer [53]. A few ncRNAs work by interacting with mRNA (antisense transcription). A mechanism in which the targeted gene is inhibited by the transcription of lncRNA from the opposite template could be a life-changing event in the treatment of hereditary disorders such as Angleman syndrome and others. Furthermore, miRNA–mRNA interactions may suppress mRNA expression. For example, the cell adhesion molecule 1 (CADM1) gene in bone cancer works by sponging miRNA, opening the way for the development of novel therapeutics [54].

## 4. Expression and Function of ncRNAs in CC

### 4.1. Dysregulated miRNAs in CC Onset/Progression

Several researchers have investigated the levels of miRNA expression in cervical carcinoma biopsies, exfoliated cervical cells, and cervical mucus, as well as in the serum of women who have been diagnosed with CC. Lui et al. reported the differential expression patterns of six miRNAs (miR-143, miR-143, miR-23b, miR-21, let-7b, and let-7c) which are unique to human CC cell lines [55]. Since then, extensive research has been carried out to characterize the mechanisms that cause miRNA dysregulation as well as profile the expression levels of miRNA in CC and normal cervical epithelial tissues. Indeed, gene knockdown, gene amplifications, or mutations in miRNA loci, coupled with epigenetic silencing such as DNA methylation or dysregulation of miRNA processors (e.g., Drosha) and transcription factors, are all attributed to abnormal miRNA expression patterns observed in cancer, including CC [56]. Muralidhar et al. uncovered 16 dysregulated miRNAs in advanced cervical SCC, including miR-203, miR-31, miR-29a, and miR-21, which have all been attributed to the overexpression of the miRNA processor (Drosha) transcripts and the acquisition of chromosome 5p [57]. Gupta et al. revel that miR-34a or miR-16 may regulate senescence, autophagy, apoptosis, and the functional G1/S checkpoint. Individually, miR-449a may influence senescence and apoptosis and coordinate autophagy in HeLa cells in a synergistic way with miR-16 and/or miR-34a [58]. Multiple studies have previously been conducted to examine the expression profile of miRNAs to find that substantial changes occur during the progression from low to high grade cervical malignancies and to invasive cervical carcinoma, concerning the recognition of unique biomarkers for the determination of cancer stage as well as for resolving diagnosis and prognosis purposes [59]. Gocze et al. conducted miRNA profiling and consequently identified the upregulation of miR-21, miR-34a, miR-196a, miR-27a, and miR-221, which serves as a distinct hallmark of HPV positivity in cervical malignancy samples, regardless of the clinical tumor grade [60]. Tian et al. revealed that the use of single miR-424 and/or miR-375 detection, a miR-424/miR-375/miR-218-based multi-marker panels, is more effective than using cytology in cervical exfoliated cells in gynecological clinics for screening HPV-positive women [61]. MiR-424/miR-375/miR-34a/miR-218 exhibited a statistically significant reduction in expression in high-grade cervical intraepithelial neoplasia (CIN) and abnormal cytology compared to low-grade CIN and normal cytology [61]. Cervical mucus analysis has also been shown to be an effective technique for detecting cervical neoplastic tumors. MiRNAs (miR-20b-5p, miR-126-3p, miR-451a, and miR-144-3p) found in cervical mucus have been shown to be helpful in detecting CC and high-grade intraepithelial lesions [62]. Taken together, the discovery and development of unique tumor biomarkers in cervical exfoliated cells and biological fluids could help with cancer screening and/or reappearance monitoring after treatment. However, further research is needed to determine the clinical implications of miRNA for cancer diagnosis and prognosis.

#### 4.1.1. Oncogenic miRNAs

Evidence from prior studies shows that the overexpression of ncRNAs encourages the growth and development of cervical carcinoma cells and tissues [63,64]. Various studies have demonstrated that the dysregulation of miRNAs significantly contributes to the progression and proliferation of cancerous cells, and they play a crucial role in spreading cancer via advancing cancer growth, development, progression, invasion, angiogenesis, and metastases. miR-21 is found to be overexpressed in aggressive CC tissues, and researchers have shown that miR-21 increases the proliferative index and enhances the migratory and invasion abilities of cervical cells in HeLa cell lineages by considerably repressing the expression of the tumor-suppressive Phosphatase and tensin homolog (PTEN) gene [65,66]. Xu et al. identified that oncogenic miR-21 downregulates PTEN gene expression and increases cell proliferation and migratory and colony forming ability in invasive CC [67]. Overwhelming evidence revealed the involvement of numerous miRNAs in CC progression, as listed in Table 1.

#### 4.1.2. Tumor Suppressor miRNAs

Numerous miRNAs have been identified to be involved in suppressing different malignancies involving CC, indicating that they play a critical function as tumor suppressor miRNAs. For example, HPV-associated miR-29 acts as a tumor suppressor in CC [82]. miR-520d-5p is downregulated in CC and target PTK2 and promotes apoptosis and inhibits CC cell proliferation, migration, and invasion [83]. miR-203 is also downregulated in CC and targets VEGF, which leads to suppressed CC cell proliferation, tumor development, and angiogenesis [84].

Moreover, several studies have demonstrated that circRNA also plays a crucial role in the development and progression of cancer [5,85]. The majority of circRNAs are found in the cytoplasm and typically function as competitive endogenous RNAs (ceRNAs) by sponging miRNAs and enhancing downstream gene expression [86,87]. In a recent study, Ma et al. discovered that circRNA-000284 are upregulated in CC tissues and promotes cell proliferation and invasion in CC cells. Further, the knockdown of circRNA-000284 suppresses the proliferation and migration of CC cells by sponging miR-506 and downregulates the expression of Snail-2 [88]. Circ 0087429, which is controlled by EIF4A3, may reverse EMT and inhibit CC development through the miR-5003-3p/OGN axis and it is predicted to become a potential target for CC therapy [89]. Recently, Chen et al. found that miR-138 inhibits CC tumor growth by specifically targeting EZH2, showing that DNA methylation at the miR-138 promoter contributes to its downregulation. This study suggests that miR-138 might be used to predict CC metastasis and/or used as a therapeutic target [90]. CircLMO1 overexpression inhibited the growth and metastasis of CC cells both in vitro and in vivo whereas its knockdown increased the proliferation and invasion of CC cells. Mechanistically, CircLMO1 functioned as a competitive endogenous RNA (ceRNA) by sponging miR-4192 to inhibit target gene ACSL4, suggesting that it might be a promising biomarker for the clinical management of CC [91]. In depth research suggests that several miRNAs play a prominent role in CC suppression, many of which are listed in Table 2.

### 4.2. Dysregulated lncRNAs in CC Onset/Progression

LncRNAs possess the potential to bind proteins, mRNAs and miRNAs, and they are found to be engaged in a wide range of biological events as well as cancer formation. Numerous lncRNAs such as HOTAIR, MALAT-1, H19, CCAT2, GAS5, SPRY4-IT1 LET, CCHE1, MEG3, EBIC, and PVT1 are known to perform important roles in cervical tumorigenesis, growth, development, migration, metastases, dissemination, invasion, as well as radio-resistance [111]. NORAD, a long non-coding RNA, could be a key regulator in tumor progression. Huo et al. show that NORAD expression was observed to be significantly upregulated in CC tissues and cell lineages and promotes the development and dissemination of CC by sponging miR-590-3p and targeting SIP1. Aberrant expression of NORAD is attributed to advanced FIGO stage, vascularization lymph node metastases, and poor overall survivability of CC patients. On the other hand, silencing of its expression lowered CC cell division, incursions, and EMT processes [112].

Another lncRNA, CCHE1, was also found to be deregulated in CC and its aberrant expression was linked to a poor prognosis in CC patients, indicating that CCHE1 could be used as a prognostic biomarker [113]. Similarly, lncRNA CCAT2 prominently contributes to CC and it was reported that the knocking down of CCAT2 impeded cervical tumor cell proliferation and caused CC cells to enter the G1 phase of their cycle and stimulated them to undergo autophagy [114]. Taken together, lncRNAs perform various functions and aid in the diagnosis, treatment, and prognosis of CC. However, further studies are required to provide a better understanding.

#### 4.2.1. Oncogenic lncRNAs

Evidence from prior findings reveals that the dysregulation of lncRNA influences the growth and development of CC cells and tissues. Several lncRNAs have been involved in the cancer progression such as HOX transcript antisense intergenic RNA (HOTAIR), H19, and X-inactive specific transcript (XIST), plasmacytoma variant translocation 1 (PVT1), cervical carcinoma high-expressed 1 (CCHE1), and metastasis-associated lung cancer adenocarcinoma transcript 1 (MALAT-1) (Table 3). MALAT-1 has been demonstrated to produce epigenetic modifications and affect gene expression, nuclear organization, and alternative splicing regulation by functioning as a splicing factor decoy [115]. MALAT-1 is expressed exclusively in cervical carcinoma cell lineages and tumor tissues contaminated with HR-HPV [116]. It functions by sponging numerous miRNAs, such as miR-145, and thus encourages the development and progression of cervical carcinoma through the induction of EMT [116].

#### 4.2.2. Tumor Suppressor lncRNAs

In CC, a few lncRNAs act as tumor suppressors (Table 4). Maternally expressed Gene 3 (MEG3) is a well-recognized suppressive lncRNA that increases apoptosis and suppresses the multiplication of CC cells through specifically linking with p-STAT3 and consequently causes its ubiquitination and destruction [135]. In other study, STXBP5-AS1 lncRNA is also downregulated in CC. STXBP5-AS1 decreases the invasion and migration ability of cervical cancer cells via miR-641/PTEN axis [136].

## 5. Role of ncRNAs (miRNAs and lncRNAs) in the Tumor Microenvironment (TME) of CC Onset/Progression

TME is a complex and dynamic network composed of tumor cells and their surroundings, which includes tumor-linked immune cells, vascular endothelial cells, fibroblasts, pericytes, adipocytes, extracellular matrix (ECM), cytokines, and chemokines [146]. The ECM and various types of stromal cells comprise the TME. Crosstalk between tumor cells and their TME is a crucial event in tumor progression and metastasis [147]. The emerging data suggest that ncRNAs (miRNA and lncRNA) play a significant role in modulating TME as well as tumor progression [148]. However, more research is needed for a better understanding of the physiological and pathological functioning of ncRNAs in the TME.

Matrix metalloproteinases (MMPs) are extracellular proteinases that have an impact on primary tumor invasion and metastasis. Clinical studies in CC indicated that miR-183 decreases CC cell proliferation and metastasis by inhibiting MMP-9 [149]. Similarly, activation of angiogenesis is required for solid tumor development and metastasis in TME [150]. In CC cells, miR-124 has been shown to target the angiomotin-like protein AmotL1 and subsequently decrease clonogenicity and cellular proliferation [99]. Additionally, cervical squamous cancer cells release exosomal miR-221-3p, which has been demonstrated to facilitate angiogenesis via targeting Thrombospondin-2 [151].

Similarly, lncRNAs have been demonstrated to facilitate crosstalk between tumor cells and stromal cells, and the deregulation of their expression in these cells might result in carcinogenesis [152]. The lncRNA MALAT-1 (metastasis-associated lung adenocarcinoma transcript 1), for example, is substantially expressed in patients suffering from non-small-cell lung cancer (NSCLC), and that of exosomal MALAT-1 is linked to the tumor, node, and metastasis (TNM) stage [153]. The lncRNA can also help tumor cells evade immune recognition by promoting the formation of an immunosuppressive microenvironment [154]. Collectively, tumor onset, progression, dissemination, metastasis, and other malignant biological characteristics can all be influenced by information exchange in the TME [155]. Clinically, ncRNA-mediated modulation of the TME and crosstalk between cancer and immune cells has emerged as a promising and appealing diagnostic and therapeutic.

## 6. Role of ncRNAs (miRNAs and lncRNAs) in the Tumor Immunology of Onset/Progression

The immune system is well acknowledged for its participation in cancer onset and development, and it can have both pro-carcinogenic and anti-carcinogenic effects contingent on the microenvironment [156]. The adaptive immune system provides highly specialized procedures that eliminate pathogens, while the innate immune system is the initial line of defense against foreign pathogens [157]. Importantly, the immune system can kill cancer cells in addition to defending against foreign invaders. In recent decades, researchers and physicians have focused on effectively activating the immune system to better combat cancer, and this treatment is referred to as “immunotherapy”. Due to its exceptional and long-lasting efficacy, immunotherapy has been designated as the fourth treatment cornerstone of cancer therapy [158]. However, only a small proportion of patients benefited from immunotherapy. The data suggest that ncRNAs are active participants in several stages of tumor immunity. NcRNAs, which include miRNAs, lncRNAs, and circRNAs, influence a wide range of cellular activities in the development and progression of cancer [159].

A better understanding of the function of ncRNA in the control of cancer immunity will lead to the development of novel treatment targets. Therefore, extensive research is needed to understand the function of ncRNAs in cancer immunity and obtain new insights into cancer diagnostics and immunotherapeutic therapy. Effector cells, such as macrophages, natural killer (NK) cells, and neutrophils, are crucial components of the innate immune response [157]. NcRNAs play a crucial role in the regulation of these effector cells. For example, enforced expression of miR-511-3p, has been shown to suppress tumor formation by downregulating the protumoral gene profile of mannose receptor-1 (MRC1)^+^ tumor-associated macrophages (TAMs) [160]. Several research studies have been undertaken to determine the role of ncRNAs in macrophage polarization since it is a critical component of many disease states, including cancer [158]. TCONS_00019715, an lncRNAs, play a key role in driving macrophage polarization to the M1 phenotype, which improves tumoricidal capabilities [159]. In addition, miRNA-19a-3p, miR-33, and lncRNA-MM2P impact M2 macrophage polarization [161]. MiR-21 regulates colony-stimulating factor 1 receptor (CSF-1R) for macrophage repolarization [162], whereas a double feedback loop regulated by miRNA-23a/27a/24-2 effectively regulates macrophage polarization and regulates cancer progression [163]. Surprisingly, the roles of macrophages in tumors must be contextualized within the unique microenvironment since macrophages exhibit intensities of cytokines, hence serving as either anti-carcinogenic or pro-carcinogenic [164]. NK cells have anti-cancerous properties, and it was shown that ncRNAs play a significant role in NK cell biology in the domains of growth, inflammation, and tumor monitoring [165]. He et al. revealed that the presence of various miRNAs in circulation, such as miR-122, miR-21, miR-15b, and miR-155, can stimulate NK cells via Toll-like receptor signaling and inhibit tumor formation [166]. Evidence suggests that ncRNAs play a critical role in adaptive immunity and influence tumor development and dissemination. For example, miRNA let-7a expression in colorectal cancer tissue may be negatively correlated to T-cell density and positively associated with colorectal cancer cell death [167]. Hui et al. recently identified and validated six immune-related lncRNAs (AC006126.4, EGFR-AS1, RP4-647J21.1, LINC00925, EMX2OS, and BZRAP1-AS1) of CC and revealed an immune-related risk model for predicting clinical outcomes, indicated the intensity of immune cell infiltration in the TIME, and predicted potential compounds in the immunotherapy treatment for CC [168].

## 7. Role of ncRNAs (miRNAs and lncRNAs) in Cancer Stem Cells (CSCs) of CC

Despite HPV infection being the most common cause of CC, CSCs also play an important role in the disease’s development, metastasis, recurrence, and prognosis [169]. CSCs play a significant role in the recurrence and metastasis of patients with cervical carcinoma [170,171]. Several recent studies have reported that the stemness properties are partly regulated by the interaction of ncRNAs in CC stem cells.

In recent research, Xia et al. found that AFAP1-AS1 suppresses cancer stemness, cell cycle progression, and EMT in CD44v6 (+) CC cells, and that the miR-27b-3p/VEGF-C axis is a direct target of AFAP1-AS1, allowing AFAP1-AS1 to modulate stemness characteristics in CC cells [172].

Another study suggests that urothelial carcinoma-associated 1 (UCA1) is a lncRNA with aberrant expression in a number of malignant tumors [173]. There has been less research on the involvement of UCA1 from CC cell-derived exosomes in CC development. UCA1 overexpression reduces the cytoplasmic levels of free miR-122-5p, reducing miR-122-5ps ability to regulate its target mRNAs [174]. Another study also supports that CaSki- exosomes can influence CC stem cell self-renewal and differentiation, but silencing UCA1 or increased expression of miR-122-5p inhibits CC stem cell self-renewal and differentiation [175].

Transcription factor 4 (TCF-4) is a transcription factor that interacts with β-catenin to activate target gene transcription in response to Wnt activation signaling [176]. The uncontrolled activation of the smoothened (Smo) signal transducer of the oncogenic Hedgehog (Hh) pathway in chronic myeloid leukemia has been linked to the downregulation of miR-326. Restoring miR-326 expression may also aid in the elimination of CD34 + CML stem/progenitor cells [177]. In patients receiving CSC-targeted treatment, CD133 might be used as a specific CC stem cell marker [170]. Zhang et al. reveal that the overexpression of miR-326 significantly decreased TCF-4 protein expression. Furthermore, miR-326 inhibited CaSki cell growth and CSC-like properties in vitro by targeting TCF-4 [178].

Human bone marrow mesenchymal stem cell (hBMSCs)-derived extracellular vesicle (EVs)-loaded miR-144-3p altered the biology of recipient CC cells by curbing cell proliferation, migration, invasion, and clonogenicity while inducing apoptosis, all of which lead to a decreased propensity in the development and progression of CC [179]. Another study reported that miR-135a triggered the development of a CD133+ subpopulation in an HPV-immortalized cervical epithelial cell line. In both in vitro and in vivo studies, miR-135a induced the formation of a subpopulation of cells with CSC characteristics, and the Wnt/β-catenin signaling pathway is required to maintain its tumorigenicity [180]. Dong et al. show that miR-146a downregulation promotes tumorigenesis in CC stem cells via the VEGF/CDC42/PAK1 signaling pathway [181].

CC is often related to HPV infection and the HPV 16 E5 gene has been shown to promote EGFR expression by blocking the degradation of internalized EGFR [13], and HPV 16 E6/E7 has also been demonstrated to increase EGFR levels [182]. The study reports on the link between let-7i-5p, miR-181a-2-3p, and the EGF/PI3K/SOX2 axis, which is essential for the survival of CSCs in CC, Let-7i-5p, miR-181a-2-3p, or SOX2, could be possible treatment targets for cervical CSCs, if more research is carried out on CC tissue samples and in vivo [183].

Several cancers express the homeobox A11 antisense lncRNA (HOXA11-AS), which is near the HOXA11 gene, supporting the concept that it promotes CC progression [184]. NcRNAs dominate homeobox gene cluster intergenic transcripts, which comprise short miRNA and lncRNAs that are antisense to their conventional HOX neighbors. HOX transcription factors promote embryonic development in both humans and mice [185]. In vitro, HOXA11-AS overexpression increased cell proliferation, migration, and tumor invasion, whereas HOXA11-AS knockdown decreased these biologic aggressive characteristics [186]. HOTAIR functions as an oncogenic lncRNA and plays a critical role in regulating stemness properties in various cancers, including CC [187,188]. HOTAIR is significantly elevated in association with the enrichment of CC stem cells, and its knockdown dramatically reduces the expression of stemness markers. The level of HOTAIR was found to be linked to the expression of miR-203, which helps EMT and is controlled by ZEB1 [189].

## 8. Therapeutic Approaches for Targeting ncRNAs in CC

The use of ncRNAs as a therapeutic target for CC might be very effective. Antisense oligonucleotides (ASOs), the CRISPR–Cas9 system, exosomes, and other methods are currently being used to exploit the therapeutic value of lncRNAs.

ASOs are single-stranded antisense oligonucleotides having a central DNA stretch (>6mers) which can be native or phosphorothioated (chemically modified), and RNA nucleotides at flanking sections of the molecule [190]. Several diseases have been successfully treated with ASOs, which are often employed to change mRNA expression [191,192]. They may be used to inhibit cancerous ncRNAs that are overexpressed in cancer cells. A recent study has shown that LncRNA MALAT-1-specific ASOs suppress cancer cell metastasis in vitro and in vivo [193]. CC therapy based on ASOs needs further investigation at this point. As antisense oligonucleotide technology continues to evolve, research into the clinical use of ASO as a therapy for CC is likely to move quickly.

Therapeutic targeting of coding and non-coding genes is now possible using the CRISPR–Cas9 system [194,195]. There are many ways to employ this system: genome editing using active CRISPR–Cas9 or Cas12a (Cpf1), interfering with gene activity or activation using catalytically dead (d)Cas9 linked to an activating or repressive effector domain, or RNA editing using the Cas13 variant [196,197,198]. Ex vivo CRISPR–Cas9 genome editing clinical studies are now underway. Several lncRNAs have been shown to either promote (SAF, MALAT-1, HEAL) or inhibit (GAS5, 7 SK, NRON, TAR-gag, lincRNA-p21, NEAT1). [199]. Viral proteins can also modify the biological activities of lncRNAs through direct or indirect binding, hence altering their protein and/or nucleic acid interactomes. For instance, HPV16 E7 has been shown to communicate with HOTAIR, potentially impairing its ability to suppress polycomb-regulated genes [200]. The lncRNA urothelial carcinoma-associated 1 (UCA1) was recently shown to be critical in human heme biosynthesis and erythrocyte development of CD34 + HSCs. In this study, Liu et al. identified that lncRNA UCA1 serves as a scaffold for recruiting PTBP1 to ALAS2 mRNA and stabilizing it through PTBP1 [201]. This demonstrates that lncRNAs may increase the number of therapeutic CRISPR–Cas9 ex vivo editing targets [201]. Delivery concerns, particularly with the big Cas proteins, must be resolved before the CRISPR–Cas9 system can be used in vivo, and immunological responses must be carefully examined. In spite of its numerous benefits, the CRISPR–Cas9 system may have detrimental impacts owing to off-target effects that cannot be ignored and might have major implications [202]. The CRISPR–Cas9 technology has several constraints that need to be solved in order to enhance its therapeutic use.

In addition, exosomes are nanovesicles that facilitate communication between cells. Exosomes are important in the development and progression of cancer [203]. Exosomes include functional components such as proteins, lipids, mRNA molecules, and ncRNAs which act as carriers of extracellular information [204]. With the advancement of scientific technology, we expect that exosomes encapsulating lncRNAs or specialized drugs targeting lncRNAs will be developed for cancer-targeted treatment.

## 9. Approaches for Systemic Delivery of Therapeutics ncRNAs in CC

Ensuring ncRNA therapeutics reach their intended target organ and cell type, as well as cross cell membranes to accomplish their intracellular activities, is one of the major hurdles in the field. Oligonucleotide delivery is limited by its instability, negative charge, and hydrophilic nature, which hinders diffusion across cell membranes [205]. Lipid and polymer-based vectors, as well as ligand–oligonucleotide conjugate delivery systems, are all being employed as delivery methods. Endosomal escape of the RNA therapy must be made easier to avoid lysosomal degradation because of the variety of endocytosis mechanisms used to pick up these delivery systems.

Lipid nanoparticles (LNPs) are readily manipulated, may be linked to targeting moieties, and have great biodegradability and biocompatibility with low immunogenicity. The elevated lncRNA ceruloplasmin (NRCP) was suppressed in an ovarian cancer mouse model utilizing a phosphocholine-derived 1,2-dioleoyl-sn-glycero-3-phosphocholine (DOPC) nanoliposome carrying siRNA. There was a significant decrease in tumor development and greater sensitivity to cisplatin [206].

Polymer-based carriers differ from lipid-based ones in that they are more versatile in terms of size, molecular composition, and structure. Several polymers, including polyethylene imine (PEI), polylactic-co-glycolic acid (PLGA), poly-amidoamine (PAMAM), and chitosan, have been extensively investigated for the delivery of miRNA mimics or antimiRs, alone or in combination with chemotherapies for improved therapeutics. Several miRNAs such as miR-150, miR-221/miR-222, miR-21, miR-34a, miR-145, and miR-33a have all been delivered systemically or locally through polymers [207,208,209,210,211].

LOcal Drug EluteR (LODERTM) is a new biodegradable polymeric matrix that protects drugs from enzymatic degradation and releases siRNA against G12D-mutated KRAS (siG12D), and a phase 1/2a clinical study of siG12D-LODER was recently completed [212]. Combinatorial therapies can potentially benefit from the development of nanoparticles (NPs) that can deliver multiple therapies at once, and a lot of progress has been made in this area [213].

The oligonucleotide conjugation of diverse entities is being further investigated for their delivery. Ligand conjugation is a frequent clinical strategy for RNA therapeutic delivery to cancer, allowing selective administration through a receptor-mediated mechanism [214]. A recent study showed that the inhibition of the ubiquitin-conjugating enzyme E2 N by miRNA-590-3p reduced the cell growth of CC [215]. Furthermore, in an A549 xenograft model, T7-conjugated Co-ASOs-LNPs (Co-ASOs-LNPs) displayed improved anticancer efficacy, prolonged overall survival time, and tumor targeting activity [216]. Another promising preclinical strategy is conjugation of oligonucleotides to antibodies gates. In this technique, oligonucleotide or drug may be conjugated to antibodies through electrostatic interactions, affinity conjugation using biotin or avidin, direct conjugation, or double-strand hybridization [217,218]. Several alternative approaches, such as using an azide-functionalized linker peptide on the antibody and conjugation to dibenzylcyclooctyne-bearing RNAs or antibodies with a reactive lysine residue paired with β-lactam linker-functionalized RNAs, have also been investigated [219,220].

Numerous advances have been achieved in this sector, which is especially relevant for combinatorial therapies, such as the development of NPs.

By optimizing the targeted delivery of medicines specifically to tumor regions coupled with enhanced efficiency, nanomedicine offers the potential to overcome the limits of traditional therapeutic techniques [221]. The application of NPs as a ncRNA-targeted treatment coupled with immunotherapy seems feasible. However, only a few studies have been carried out to evaluate the use of this delivery system and it will take a while to implement this clinically. Shao et al. successfully developed floral-shaped SiO–PEI NPs which have maximum loads of pDNA/siRNA. These NPs containing a plasmid-expressing miR-let-7c-5p were effective in transferring miR-let-7c-5p to human epithelial cancerous HeLa cells. Furthermore, under relatively low cytotoxic situations, the collaboration of nanotechnology with gene therapy may prevent the onset and progression of cancer. Findings from this study have provided a new anticancer strategy [222]. Similarly, Wang and Liang synthesized a conjugate containing CD59, miRNA-1284, and cisplatin (CDDP), which was subsequently loaded into liposomes (CD/LP-miCDDP). This co-delivery strategy had greater anticancer effects in CC cells, and the apoptosis rate was significantly increased compared to miR-1284 or cisplatin or alone [223]. Similarly, in other cancers, nanomedicines have given good outcomes. For example, to treat lung cancer, Gong et al. efficiently synthesized MALAT-1-targetted ASOs and nucleo-targeted Tat peptide integrated with Au NPs (i.e., ASO-Au-Tat NPS), which might stabilize friable ASOs, improve nuclear uptake, and exhibit excellent biocompatibility. MALAT-1 expression in A549 lung cancerous cells was dramatically reduced after treatment with ASO-Au-Tat NPs [193].

## 10. Concluding Remarks

Although CC is a curable disease with proven interventions, it remains the most frequently occurring cancer in women globally. Individuals with advanced or metastatic CC have a very poor prognosis and available treatment options are also limited. As a result, it is critical to gain insight into the mechanisms of metastasis and identify new therapeutic targets. The incredible and sophisticated underlying molecular mechanisms that orchestrate life’s fundamental concepts are now known to be controlled by a world of highly complex non-coding RNA. The effective implementation of RNA-based therapeutics necessitates a novel multidisciplinary strategy that includes technological advances in molecular biology, immunology, pharmacology, chemistry, and nanotechnology. Considering their active participation in various pathways of cervical tumorigenesis, research on ncRNAs has emerged as a focal point for expanding our knowledge of cancer biology and offering additional research opportunities. An ideal RNA therapeutic should be rigorously assessed for immunogenicity, chemically altered to improve pharmacokinetics and pharmacodynamics, analyzed for biodistribution and potential intracellular escape mechanisms, target specificity and interactions, and be dosed at optimum concentrations to yield desired outcomes. Repeated attempts to divulge the functions of all types of ncRNAs in tumor immunity will lay the foundation for an even better understanding, control, and cancer therapy, as well as make immunotherapy more coherent with an individual’s biological properties. Additionally, modifying the tumor microenvironment can provides striking results in the prevention and pathological management of CC, as evidenced by the current report of clinical trials in oncology. Successfully prepared nanomedicines bring a massive shift and ongoing clinical research in oncology demonstrates that nanoscience will shortly provide unique therapeutic approaches for thousands of CC patients globally.

## Figures and Tables

**Table 1:** Summary of selected oncogenic miRNAs with their functional effects in CC.

MiRNAs	miRNA Expression Profile	Target Gene/Pathway	Biological Function of Oncogenic miRNAs	Ref.
miR-17-5p	Upregulated	TGFBR2	Promotes CC cell metastasis and proliferation	[68]
miR-9-5p	Upregulated	SOCS5.	Promotes angiogenesis, cell proliferation, and invasion	[69]
miR-96	Upregulated	CAV-1	Promotes cell proliferation, migration, and invasion	[70]
miR-21	Upregulated	RASA1	Promotes metastasis and enhances the invasiveness of CC cells	[71]
miR-221-3p	Upregulated	TWIST2/THBS2	Promotes metastases of the lymph nodes in CC	[72]
miR-199 b-5p	Upregulated	KLK10	Promotes cell proliferation, migration, and inhibits apoptosis	[73]
miR-106b	Upregulated	DAB2/TGF-β1	Induces migration of CC cells	[74]
miR-519d	Upregulated	Smad7	Promotes invasiveness and migration abilities of CC cells and prevent cell autophagy	[75]
miR-378	Upregulated	ATG12	Promotes metastases and inhibits apoptosis	[76]
miR-20a	Upregulated	TIMP2 and ATG7	Increases histopathological grade, tumor size, and distant metastases	[77]
miR-106a	Upregulated	TIMP2	Promotes the cell migration and invasion	[78]
miR-150	Upregulated	PDCD4	Promotes cell invasion and migration	[79]
miR-31	Upregulated	BAP1	Promotes cell proliferation and modulates the EMT	[80]
miR-155	Upregulated	LKB1	Promotes CC cell proliferation	[81]

**Table 2:** Summary of selected tumor suppressor miRNAs with their functional effect in CC.

MiR-RNA	Expression Pattern of miRNAs	Target Gene/Pathway /Molecule	Biological Function of Tumor Suppressor miRNAs	Ref.
miR-520d-5p	Downregulated	PTK2	Promotes apoptosis and inhibits CC cell proliferation, invasion, and migration	[83]
miR-125	Downregulated	VEGF and PI3K/AKT	Inhibits CC cell growth and tumor progression	[92]
miR-23b	Downregulated	AKT/mTOR	Inhibits CC cell multiplication invasion and migration abilities	[93]
miR-29a	Downregulated	DNMT1-SOCS1/NF-κB	Inhibits proliferation, migration, and invasion and promotes CC cell apoptosis	[94]
miR-543	Downregulated	P13K/AKT, p38/MAPK and TRPM7	Inhibits cell proliferation, migration, and invasion; induces cell cycle arrest and boost apoptosis	[95]
miR-497	Downregulated	IGF-1R	Inhibits cell proliferation and arrest cells at S phase of cell cycle	[96]
miR-218	Downregulated	Survivin (BIRC5)	Inhibits clonogenicity, invasion, and migration	[97]
miR-200b	Downregulated	Rho-E	Inhibits migration potential of CC cells and therefore their ability to metastasize	[98]
miR-124	Downregulated	AmotL1	Inhibits angiogenesis, migration, and invasion	[99]
miR-214	Downregulated	EZH2	Inhibits proliferation of CC cells	[100]
miR-203	Downregulated	VEGFA	Inhibits cell proliferation, tumor development, and angiogenesis	[84]
miR-143	Downregulated	Bcl-2	Inhibits cell proliferation and promoted apoptosis	[101]
miR-101-5p	Downregulated	CXCL6	Inhibits colony formation, invasion, and migration	[102]
miR-132	Downregulated	SMAD2	Inhibits lymph node metastasis	[103]
miR-129-5p	Upregulation	ZIC2	Inhibits tumorigenesis and angiogenesis	[104]
miR-138-5p	Downregulated	SIRT1	Inhibits the tumorigenesis and metastasis	[105]
miR-142-3p	Downregulated	CDC25C	Inhibits cell proliferation	[106]
miR-148b	Downregulated	CASP3	Inhibits cell proliferation and promoted apoptosis	[107]
miR-182	Downregulated	DBMT3a	Induces apoptosis and inhibits cell proliferation	[108]
miR-195	Downregulated	Smad3	Inhibits cell proliferation, migration, and invasion	[109]
miR-196b	Downregulated	VEGF	Inhibits angiogenesis	[110]

**Table 3:** Summary of selected oncogenic LncRNAs with their functional effects in CC.

LncRNA	Expression Pattern lncRNA	Target Gene /Pathways/Molecules	Biological Function of Oncogenic lncRNA	Ref.
HOTAIR	Upregulated	BCL2, miR-143-3p	Promotes CC cell growth	[117]
LINC01535	Upregulated	miR-214/EZH2 feedback loop	Promotes progression and metastasis of CC	[118]
CDKN2B-AS1	Upregulated	miR-181a-5p/TGFβI axis	Promotes tumor cell growth and inhibits apoptosis	[119]
CASC11	Upregulated	Wnt/β-catenin	Promotes cell proliferation	[120]
LINC00675	Upregulated	Wnt/β-catenin	Promotes cancer cell growth, invasiveness, migration, and repressed cell apoptosis	[121]
MALAT-1	Upregulated	HPV16 E6/E7	Promotes cell proliferation, migration, and invasion and modulates EMT expression	[122]
ANRIL	Upregulated	Cyclin D1, CDK4, CDK6, E-cadherin, vimentin, and N-cadherin.	Promotes cell proliferation, migration, and invasion and inhibits apoptosis	[123]
BLACAT1	Upregulated	Cyclin B1, and CDC25C, N-Cadherin, E-Cadherin	Enhances CC cell proliferation and invasion	[124]
PVT1	Upregulated	Smad3, miR-140-5p sponging	Promotes cell proliferation and metastasis	[125]
HOXD-AS1	Upregulated	Ras/ERK,	Enhances cell proliferation, migration, and invasion	[126]
DLX6-AS1	Upregulated	miR-16-5p/ARPP19 axis	Increases cell proliferation and invasion	[127]
CRNDE	Upregulated	PI3K/AKT	Promotes cell proliferation and inhibits apoptosis	[128]
CCAT2	Upregulated	Cell cycle	Promotes cell multiplication and penetration	[114]
EBIC	Upregulated	EZH2, E-cadherin	Promotes metastasis and invasion	[129]
RSU1P2	Upregulated	IGF1R, N-myc, let-7a, EphA4	Promotes tumor development	[130]
SPRY4-IT1	Upregulated	miR-101-3p, ZEB1	Promotes cell proliferation, migration, and invasion and modulates EMT expression	[131]
NEAT1	Upregulated	miR-377/FGFR1 axis	Increases CC cell survival and motility and inhibits apoptosis	[132]
FAM83H-AS1	Upregulated	E6-p300 pathway	Promotes cell proliferation and migration and inhibits apoptosis	[133]
C5orf66-AS1	Upregulated	miR-637/RING1 axis	Promotes progression and proliferation of CC cells	[134]

**Table 4:** Summary of selected tumor suppressor lncRNAs with their functional effects in CC.

LncRNAs	Expression Pattern	Target Genes /Pathways/Molecule	Biological Function of Tumor Suppressor lncRNA	Ref.
MEG3	Downregulated	p-STAT3	Inhibits cell proliferation and increases apoptosis	[135]
GAS5	Downregulated	miR-205, miR-196a	Inhibits growth and metastases	[137]
GAS5-AS1	Downregulated	Increase GAS5 stability by epigenetic modulation	Suppresses growth and metastasis	[138]
STXBP5-AS1	Downregulated	miR-96-5p/PTEN axis	Inhibits cell proliferation and invasiveness of CC cells	[136]
TUSC8	Downregulated	miR-641/PTEN axis	Inhibits migration and invasion	[139]
XLOC_010588	Downregulated	c-Myc	Inhibits proliferation	[140]
LINC00861	Downregulated	PTEN/AKT/mTOR miR-513b-5p	Inhibit the progression of CC cells	[141]
ZNF667-AS1	Downregulated	Sponge miR-93-3p and upregulate PEG3	Inhibits cell proliferation, invasion, and metastasis	[142]
RP11-284F21.9	Downregulated	PPWD1, miR-769-3p	Inhibits cell proliferation, migration, and invasion	[143]
Lnc-CCDST	Downregulated	DHX9-MDM2	Inhibits angiogenesis and invasion	[144]
DGCR5	Downregulated	WNT signaling	Suppresses migration and invasion	[145]

## Data Availability

Not applicable.
